# Aberrant expression of MAPK1 and MCTS1 in chronic myeloid leukemia (CML)

**DOI:** 10.17912/micropub.biology.001681

**Published:** 2025-09-18

**Authors:** Leo Kortendick, Corinna Meyer, Stefan Nagel

**Affiliations:** 1 Human and Animal Cell Lines, Leibniz Institute DSMZ – German Collection of Microorganisms and Cell Cultures, Braunschweig, Lower Saxony, Germany

## Abstract

Genomic amplification may result in aberrant gene expression and support development of cancer, including chronic myeloid leukemia (CML). In CML cell line K-562, we recently reported overexpression of TBX1 located at chromosomal position 22q11, focally co-amplified together with BCR, part of the CML hallmark fusion gene BCR::ABL1. Here, we extended that study, by identifying genomically amplified and overexpressed MAPK1/ERK2 at 22q11 together with MCTS1 at Xq22. Using pharmacological inhibitors and siRNA-mediated knockdown assays, our data collectively revealed novel regulatory connections between TBX1, MAPK1 and MCTS1, which may play a role in drug resistance.

**Figure 1. Deregulation of MAPK1 and MCTS1 in CML:**
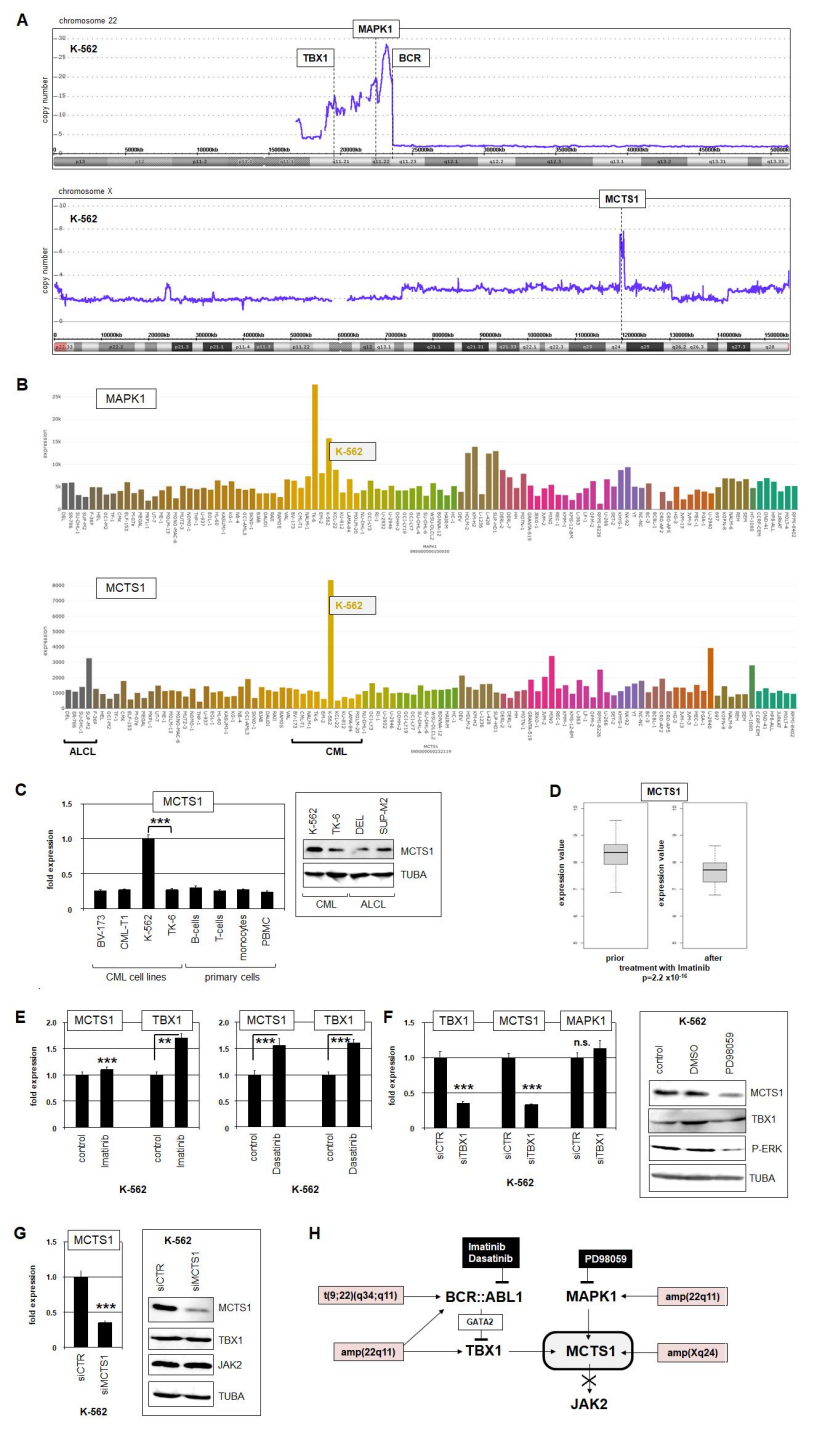
(A) Genomic copy number data from K-562 indicated an amplicon at 22q11, targeting TBX1, MAPK1, and BCR (above), and at Xq21, targeting MCTS1 (below). (B) RNA-seq data from 100 leukemia/lymphoma cell lines showed high expression levels of MAPK1 (above) and MCTS1 (below) in CML cell line K-562. Anaplastic large cell lymphoma (ALCL) and CML cell lines are indicated. (C) RQ-PCR (left) and Western blot analysis (right) demonstrated elevated expression of MCTS1 in K-562 cells as compared to selected primary immune cells and control cell lines. (D) Analysis of public dataset GSE44589 showed MCTS1 expression in CML patients priorto and after treatment with BCR::ABL1-inhibitor Imatinib. Treated patients expressed significantly lower levels. (E) RQ-PRC analysis showed that treatment of CML cell line K-562 with BCR::ABL1 inhibitors Imatinib (left) or Dasatinib (right) activated expression of MCTS1 and TBX1. (F) RQ-PCR analysis of TBX1, MCTS1, and MAPK1 in K-562 treated for siRNA-mediated knockdown of TBX1 (left). Treatment of K-562 with ERK-inhibitor PD98059 resulted in reduced levels of MCTS and phosphorylated ERK (P-ERK) but not of TBX1, as indicated by Western blot analysis (right). (G) RQ-PCR (left) and Western blot analysis (right) confirmed MCTS1 knockdown in K-562. This treatment showed no effect on TBX1 or JAK2 protein expression levels. (H) Graphical summary of the results from this study, showing that chromosomal aberrations and overexpression of TBX1 and MAPK1 mediate MCTS1 activation, which does not impact JAK2 in CML.

## Description

Genomic aberrations, including copy number alterations and chromosomal translocations, represent frequent oncogenic mechanisms in carcinogenesis. The fusion gene BCR::ABL1 is generated by translocation t(9;22)(q34;q11), representing a hallmark for the hematopoietic malignancy chronic myeloid leukemia (CML) (Jabbour et al., 2024). BCR::ABL1 encodes a constitutively activated tyrosine kinase which drives important oncogenes and pathways, including the transcription factors STAT3 and STAT5 (Carlesso et al., 1996). This kinase is therapeutically targeted by inhibitors like Imatinib and Dasatinib, which revolutionized the treatment of this fatal disease. However, due to the appearance of resistant mutations, novel treatment options are required (Jabbour et al., 2024).

To reveal novel oncogenes and alternative treatment targets in CML, we used model cell line K-562 and analyzed genomic aberrations therein. Recently, we reported for K-562 amplification of the fusion gene BCR::ABL1 (Nagel et al.,2019), and identified co-amplification of T-box gene TBX1 at 22q11, deregulating proliferation, apoptosis and differentiation (Nagel et al.,2024).

Here, we screened genomic profiling data of K-562 for additional promising oncogenes. Within the amplicon at 22q11 we detected MAPK1 (alias ERK2), which encodes a kinase of the MAPK signalling-pathway ( **Fig. 1A** ). Furthermore, we detected a focal amplification at Xq24 targeting MCTS1, encoding a translation factor ( **Fig. 1A** ). Analysis of public RNA-seq data from 100 leukemia/lymphoma cell lines showed their conspicuous overexpression in K-562 ( **Fig. 1B** ).

We focused on MCTS1, which reportedly activates protein expression of the kinase JAK2 in T-cells (Bohlen et al., 2023). JAK2 is frequently overexpressed in cancer cells, activating the transcription factors STAT3 and STAT5, which play an oncogenic role in CML (Carlesso et al., 1996). RQ-PCR and Western blot analysis confirmed enhanced MCTS1 expression in K-562 as compared to primary immune cells and control cell lines ( **Fig. 1C** ). Analysis of public CML patient data showed decreased MCTS1 expression after treatment with Imatinib ( **Fig. 1D** ), while MAPK1 expression showed no difference. However, treatment of K-562 with Imatinib or Dasatinib resulted in elevated expression of MCTS1 in addition to TBX1 ( **Fig. 1D** ). These results show that Dasatinib is more potent than Imatinib and recapitulate the reported effect on TBX1, which operates via GATA2 (Nagel et al.,2024). Consistently, siRNA-mediated knockdown of TBX1 in K-562 resulted in downregulation of MCTS1 while MAPK1 expression did not change ( **Fig. 1E** ), demonstrating that TBX1 activates MCTS1. To analyze a potential impact of MAPK1 on MCTS1, we treated K-562 with ERK-inhibitor PD98059. This treatment resulted in reduced phosphorylation of ERK and reduced expression of MCTS1, while TBX1 remained unchanged ( **Fig. 1E** ). Thus, MAPK1 activity enhanced MCTS1 expression, which may be related to the described role of MAPK1 in development of drug resistance in CML (Härtel et al., 2012). However, siRNA-mediated knockdown of MCTS1 showed no effect, neither on JAK2 nor TBX1 protein levels ( **Fig. 1F** ), indicating that the reported regulatory connection in T-cells is not significant in CML cells.

Taken together, our data revealed an aberrant regulatory network, containing TBX1, MAPK1 and MCTS1 in CML cell line K-562 ( **Fig. 1G** ). While excluding JAK2 as downstream target of MCTS1 in K-562, MCTS1 may play a role in drug resistance in CML, thus representing a novel therapeutic target.

## Methods

Genomic profiling data were generated as described recently (Nagel et al.,2019). Data were analyzed using the Chromosome Analysis Suite software version 3.1.0.15 (Affymetrix, High Wycombe, UK) and copy number alterations determined accordingly.

Gene expression analyses were performed using public RNA-seq data for 100 leukemia/lymphoma cell lines (ArrayExpress dataset E-MTAB-7721) (Quentmeier et al., 2019; Koblitz et al., 2022), and public gene expression profiling data from 135 untreated and 63 Imatinib-treated CML patients (GEO dataset GSE44589).

Used cell lines have been authenticated and were obtained from the DSMZ (Braunschweig, Germany). SiRNAs directed against TBX1 and MCTS1 were obtained from Qiagen (Hilden, Germany), and transfected into the cells by electroporation as described recently (Nagel et al.,2024). Tyrosine-kinase inhibitors Imatinib (dissolved in water and used at 100 µM) and Dasatinib (dissolved in DMSO and used at 100 µM) and MAPK-inhibitor PD98059 (25 µM) were obtained from Sigma (Taufkirchen, Germany).

Real-time quantitative (RQ)-PCR analysis was performed using the Fast 7500 Real-time System and commercial buffer and primer sets (Life Technologies, Darmstadt, Germany). For normalization of expression levels, we quantified the transcripts of TATA box binding protein (TBP), and applied the delta-delta-Ct method.

Western blot analyses were performed as described recently (Nagel et al.,2024). Used antibodies were the following: MCTS1 (Genetex, Irvine, California, USA, GTX117793), JAK2 (Genetex, #GTX57670), P-ERK (Santa Cruz Biotechnology, #sc7383), TBX1 (Origene, Rockville, MD, USA, #TA347536), alpha-tubulin TUBA (Sigma, #T6199).

All treatments were performed twice and RQ-PCR analyses were performed in triplicate. Statistical significance was determined using the Student´s T-test. P-values are indicated by three asterisks when below or equal 0.001, absence of significance as not significant (n.s.).
